# Drinking the waters of Lethe: Bringing voluntary choice into the study of voluntary forgetting

**DOI:** 10.3758/s13421-023-01467-7

**Published:** 2023-09-25

**Authors:** Ryan P. M. Hackländer, Helge Schlüter, Magdalena Abel

**Affiliations:** 1 Department of Psychology, University of Hildesheim, Unversitaetsplatz 1, D–31141 Hildesheim, Germany; 2 Department of Psychology, University of Regensburg, Regensburg, Germany

**Keywords:** Directed forgetting, Free-choice, Item-method, Forgetting, Memory

## Abstract

The directed forgetting paradigm has long been used to test whether humans can voluntarily choose to forget learned information. However, to date, nearly all directed forgetting paradigms have involved a forced-choice paradigm, in which the participants are instructed about which learned information they should forget. While studies have repeatedly shown that this directed forgetting does lead to a decreased ability to later remember the information, it is still unclear whether these effects would be present if participants were allowed to, of their own accord, choose which information they wanted to forget. In two experiments here, we introduce a free-choice variety of the item method directed forgetting paradigm and show that directed forgetting effects are robust, both for instructed and voluntary forgetting. We discuss the implications of our findings for notions of voluntary forgetting and for the self-choice effect in memory.

## Introduction

*Lethe, the river of oblivion, rolls his watery labyrinth, which whoso drinks forgets both joy and grief. – John Milton*

In Greek mythology, the river Lethe was one of the five rivers of the underworld, and the spirits were usually made to drink from the river to forget their mortal lives. While drinking from the river Lethe would lead to complete oblivion of all memories, the question of interest in the current paper is whether humans have agency over what we remember and what we forget? In other words, can we choose the selective effects of drinking from Lethe? This question has been of interest to memory researchers, in part because researchers have long recognized the benefits of forgetting (Barrett & Zollman, 2009; MacLeod, 1998).

For example, one benefit of forgetting may be that it permits efficient memory updating. Being able to forget outdated and no longer relevant information in a targeted manner can reduce interference and allow a greater focus on what is currently important and relevant (Bjork, 1970). Another example is that forgetting may also support emotion regulation (e.g., Engen & Anderson, 2018). Being able to leave negative and stressful experiences behind can support emotional wellbeing and provide greater representation of positive contents in memory, thus strengthening a positive self-concept (for discussions of these and other potential benefits, see also Fawcett & Hulbert, 2020; Nørby, 2015).

### Research on voluntary forgetting

Research on the role of selective and voluntary forgetting began in earnest in the early 1970s as researchers started developing techniques specifically aimed at determining whether instructions to forget particular information indeed led to a decreased ability to later retrieve the information in question (Bjork, 1972; Epstein, 1972; MacLeod, 1998). One paradigm developed to study such forgetting is item method directed forgetting (IMDF).^1^ In this paradigm, participants are presented with unrelated information (often a series of single words or pictures) and after each piece of information are cued to either remember or forget the just-presented information. After a retention interval, participants are asked to retrieve ALL of the information that they were presented, regardless of whether they had been told to remember or forget the information. Typically, participants are able to retrieve more of the to-be-remembered information than the to-be-forgotten information (i.e., the DF effect). IMDF does not only arise on recall tests, but is also routinely found on recognition tests (Basden et al., 1993; MacLeod, 1975; for a recent meta-analysis, see also Hall et al., 2021). The effect is commonly attributed to continued rehearsal of to-be-remembered, but stopped rehearsal of to-be-forgotten information (Basden & Basden, 1996; Bjork, 1972; Woodward & Bjork, 1971). An issue of current debate is whether stopping rehearsal is an active and effortful process (e.g., Fawcett & Taylor, 2008, 2012; Fellner et al., 2020) or whether the forgetting arises more passively (e.g., Scholz & Dutke, 2019; Tan et al., 2020).

IMDF is usually described as intentional or voluntary forgetting, and thus provides evidence that humans have some control over what they remember and forget (Anderson & Hanslmayr, 2014; Bjork, 1970; Fawcett et al., 2013; Johnson, 1994; MacLeod, 1998). This conclusion, however, seems to be somewhat premature as nearly all previous research on directed forgetting has utilized what we are calling a forced-choice methodology, whereby subjects are specifically told which information to remember and which information to forget (but see Baldwin et al., 2021, for a free-choice procedure). This leaves open the possibility that forgetting of assigned information is possible, but not actual voluntary forgetting. In other words, it is still unclear if similar forgetting occurs, when we are given agency in choosing what we want to remember or what we want to forget.

### Research on free versus forced choice

Research on differences between free and forced choices is not new (Berlyne, 1957). Indeed, in the priming literature there is a history of distinguishing between free- and forced-choice tasks

(i.e., tasks in which participants can choose between different responses or button presses, and tasks in which a specific response is required). In this literature, while there are often differences in response times between free- and forced-choice tasks, with free-choice responses taking longer than forced-choice responses, the patterns of behavior are generally similar between the tasks. For example, negative compatibility effects (i.e., shorter reaction times when an irrelevant and inconsistent prime is effectively masked) can be observed for both forced and free-choice trials (see Bermeitinger & Hackländer, 2018).

Even in long-term memory research there is precedent distinguishing between information that is assigned to be learned versus information that is chosen to be learned. The self-choice effect refers to the finding that information that is chosen to be remembered is better remembered than information that is assigned to be remembered (Monty & Perlmutter, 1975; see also DuBrow et al., 2019; Kuhl & Kazén, 1994; Murty et al., 2015; Takahashi, 1992, 2002).

The original studies reporting a self-choice effect had subjects perform a typical paired-associates learning task, whereby two words were associated with each other, and the relationship between the two words was to be learned (Monty & Perlmutter, 1975; Monty et al., 1979; Perlmutter et al., 1971). Generally, half of the subjects were able to choose one of the words of the pairings, while the other half of subjects were yoked to the choices of the previous subjects (i.e., they learned the pairs that were the results of choices of a previous subject). The general pattern of findings was enhanced memory performance for those subjects who were able to make a choice (Perlmutter et al., 1971), be it for associative memory or memory of one of the alternatives in the absence of the other (Monty & Perlmutter, 1975), as long as the choice was somewhat meaningful or seemed to allow a certain measure of control (Monty et al., 1979; but see the description of Watanabe, 2001, below for self-choice effects when the choice did not allow for control over the situation).

The self-choice effect has also been found in other memory paradigms, besides paired-associates learning. For example, Kuhl and Kazén (1994) had subjects read sentences related to office activities and the planning of a birthday party. Subjects were asked to choose some of the sentences and were assigned others. With this paradigm Kuhl and Kazén also found a self-choice effect. Indeed, the self-choice effect has also been found when the choice was made independently of the to-be-remembered material. Murty et al. (2015) presented subjects with two “screens” on the computer. On forced-choice trials, one of the screens would reveal a stimulus to remember, while on free-choice trials the subjects would choose which of the two screens to open; the presented stimulus was, however, the same, irrespective of which screen was chosen. Using this procedure, Murty and colleagues found that the material revealed behind the screens was best remembered when the subjects could choose which screen to open (see DuBrow et al., 2019, for corroborating and expanded results).

Using a procedure that has similarities to IMDF, Watanabe (2001) had participants read a series of statements with three potential response options shown. For example, a statement may have read “an animal that feeds their offspring with milk,” with the response options being cat, rat, and platypus. The first manipulation concerned free versus forced choice. For some trials, one of the response options was underlined, forcing participants to select this response (irrespective of whether it fit the statement or not). On other trials, participants were instead free to choose one of the response options. A second manipulation was referred to as a cuing manipulation. In the free-cuing condition, all three of the response options were viable alternatives, as in the example above. In the constrained-cuing condition, only one of the response options was a viable alternative. For example, a statement in the constrained-cuing condition may have read “an animal that lays eggs,” with the same response options as before being cat, rat, and platypus (but only platypuses lay eggs, making this the correct response). In the free-cuing condition, and at least on free-choice trials, subjects could theoretically select any response, while in the constrained-cuing condition, only one answer should be selected if the subjects performed the task correctly.

Later, subjects were tested (with both free recall and recognition) on their memory for the response options they had seen, both the chosen and the non-chosen words. Two main findings became clear. Firstly, there was a self-choice effect, whereby subjects had better memory for the words that they were able to freely choose than the words they were forced to choose (i.e., the underlined words). This held even in the constrained-choice condition (i.e., when only one response option was viable). Secondly, and surprisingly, the benefits of choice were not limited to the “chosen” response options. Although the “non-chosen” options were not remembered as well as the “chosen” options, they, too, were remembered better in the free- than the forced-choice condition (for similar findings, see also Takahashi, 1991; Toyota, 2013).

For the purposes of the current paper, the results of the Watanabe (2001) paper can be interpreted in several ways. For one, the fact that non-chosen information in the free-choice condition was retrieved less effectively than chosen information could be seen as indirect evidence that participants have agency in forgetting the information they choose not to remember. However, the finding that the non-chosen information was remembered better in the free-choice than the forced-choice condition may seem to indicate that it is more difficult to forget information that we choose to forget than information that we are assigned to forget.

The findings from the Watanabe (2001) paper also seem to have been supported by a recent study that was focused on providing effect size estimates of the self-choice effect (Baldwin et al., 2021). This study used a meta-analytic approach, relying on 14 experiments that were conducted by the authors – the experiments were, however, not reported in the paper, but only provided on an Open Science Framework (OSF) page. Importantly, a subset of 12 experiments seems to have relied on a version of the IMDF task, with the experimenter assigning or the participants choosing what information to remember and forget. Across the different experiments, the authors, similar to Watanabe, found that the items that were chosen as to-be-forgotten were retrieved less frequently than words chosen as to-be-remembered. However, in some of the experiments the researchers also found that the to-be-forgotten items were remembered better in the free- than the forced-choice condition. In fact, in several experiments, the self-choice effect was actually driven by memory for the to-be-forgotten items.

### Current research

As summarized above, previous research on directed forgetting has mostly focused on forced-choice procedures, whereby subjects are told which information to remember and forget, but the results are often discussed as providing evidence of voluntary forgetting or forgetting with agency. Research on the self-choice effect has, however, provided either indirect (Watanabe, 2001) or direct (Baldwin et al., 2021) evidence about directed forgetting with a free-choice paradigm, but the focus of those studies was on the self-choice effect, rather than on the ability to voluntarily forget information.

Here, across two experiments, we aim to specifically investigate whether subjects can voluntarily choose which information to forget, and whether this choice leads to DF effects in the same way as do forced-choice instructions to forget (i.e., worse recall performance for forget words than for remember words). Also, we investigate whether the choice to forget something actually makes it more difficult to forget the information, as compared to being told to forget the information (as in Watanabe, 2001, and Baldwin et al., 2021). If this held true, one would expect smaller directed forgetting effects in the free-choice condition than in the forced-choice condition.

### Ethics statement

All research was approved by the local ethics committee and was conducted in line with the standards laid out in the Declaration of Helsinki. All participants participated of their own free will and provided informed consent before participating in the experiment. Furthermore, all participants were debriefed about the purpose of the deception inherent in directed forgetting experiments.

## Experiment 1

### Method

#### Participants

Ninety students from the University of Hildesheim participated in return for partial course credit. Given that we had no estimation of what the size of the effect would be, if at all present, regarding the difference between free- and forced-choice conditions, we decided to base our sample sizes on previous IMDF paradigms and aimed for 30 subjects per condition. A sensitivity analysis for two-tailed paired-samples *t*-tests using G*Power (Faul et al., 2007) showed that sub-samples of 30 subjects should enable us to detect medium-sized IMDF effects of Cohen’s *dz* = 0.53 (with *α* = .05 and 1-*β* = .80). Because a recent meta-analysis indicated that effect sizes for IMDF are usually quite large (Hall et al., 2021), we thought this was an appropriate choice. An additional sensitivity analysis for repeated-measures ANOVAs moreover suggested that an overall sample of 90 subjects would enable us to detect a small- to medium-sized within-between interaction effect of *f* = 0.17 (with *α* = .05, 1-*β* = .80, and a correlation of 0.5 among repeated measures). No participants were excluded from the study. For demographic information, see Table 1.

**Table 1:** Demographic information from participants in Experiment 1 and 2

	Experiment 1			Experiment 2
	Free-choice	Forced-choice	Yoked	
Sample size	30	30	30	30
Mean age (SD)	22.3 (3.9)	22 (2.5)	22.9 (4.1)	22.6 (4.0)
Gender F/M/D	24/6/0	23/7/0	25/5/0	26/4/0

#### Design

A 3 (condition: forced-choice, yoked forced-choice, free-choice) × 2 (item type: remember, forget) design was used. The factor condition was varied between subjects. The factor item type was varied within subjects (please note: in the case of free-choice condition, the response was not varied but chosen by participants).

### Material

Eighty words were selected from the WWN database (Lahl et al., 2009) for the study. The words were between four and eight characters long (*M* = 5.9), with medium valence (*M* = 5.2, between 3.5 and 7.5 on a 9-point Likert scale), with low arousal (*M* = 2.4, between 1 and 4.5 on a 9-point Likert scale), and not very uncommon (*M* = 5.8 pmw, all words listed as above 1 per million words used in a German language database, COSMAS II, W2:öffentlich: https://cosmas2.ids-mannheim.de:6344/cosmas2-web/menu.home.do). Of these 80, 40 were randomly chosen for the study phase for each subject (the word list can be found on the OSF page: https://osf.io/qfc2w/). In the forced-choice and the yoked forced-choice conditions, participants were cued to remember by the German word “Merken” or cued to forget by the German word “Vergessen”. In the free-choice condition, participants were prompted on each trial to indicate whether they wanted to remember or forget the previously learned word by pressing a corresponding button. For the distractor task in the retention interval, simple math problems were chosen (the math problems can be found on the OSF page: https://osf.io/qfc2w/).

The entire program was run using E-Prime 3.0 software (2016) (Psychology Software Tools, Pittsburgh, PA, USA). The run file was packaged with E-Prime Go (2020) (Psychology Software Tools, Pittsburgh, PA, USA) and could be downloaded by participants for remote participation (note that the program is only compatible with Windows operating system). Participants were instructed to complete the experiment in a quiet location without potential disturbances (e.g., cell phones). Upon completion of the experiment, participants were asked to upload the zip file containing their data to an anonymous upload folder (Academic Cloud; for exact instructions received by the participants, see the OSF page: https://osf.io/qfc2w/).

#### Procedure

After downloading the program (see above), participants were instructed to begin the experiment in a quiet location. Participants were first provided with information about the study, ethical considerations, and data protection considerations. After reading this information participants provided informed consent by means of a button press (informed consent was additionally considered to be given if subjects went through the process of uploading the data to the university’s cloud server).

Following the provision of consent, participants were informed that we were interested in the strategies that people use to remember relevant information. They were then instructed that they would see a list of words, some of which would need to be remembered. In all three conditions, subjects underwent a variant of the IMDF paradigm.

In the forced-choice conditions, participants were informed that they would be alerted, after each word, whether it should be remembered (if it was relevant for the test) or forgotten (if it was not relevant for the test). In the free-choice condition participants were informed that they would need to decide, after each word, whether it should be remembered or forgotten. The one constraint was that they were to attempt to remember roughly half and forget roughly half of the words. Following these instructions, there was a brief example (forced-choice conditions) or practice (free-choice condition) with four words presented. In the forced-choice condition participants saw a remember cue on two of the trials and a forget cue on two of the trials. In the free-choice condition participants were asked to choose to remember two and forget two of the words. After the example/practice, the instructions were repeated and the actual study phase began (the E-Prime files can be found on the OSF page: https://osf.io/qfc2w/).

##### Study phase: Forced-choice

In the study phase of the forced-choice condition, 40 words, from the possible pool of 80 words, were randomly chosen and presented to the participants in a random order. On half of the trials the words were followed by a remember cue and on the other half of the trials the words were followed by a forget cue. The order of the cues was also random, with no further conditions. Each trial began with a fixation cross presented in the middle of the screen for 1,000 ms. This was immediately replaced by the target word (presented in 48-point Calibri font in black in the middle of the screen on a grey background), which remained on the screen for 2,000 ms. The target word was immediately followed by a grey blank screen for 1,000 ms. The remember (MERKEN) or forget (VERGESSEN) cue was then presented for 2,000 ms (in the same manner as the target word). The cue was then followed by a blank screen for 1,000 ms. This sequence repeated until all 40 words had been presented.

##### Study phase: Free-choice

The study phase for the free-choice condition was identical to that of the forced-choice condition, with the following exception. Rather than being instructed to remember or forget a study word, participants were given the choice to remember or forget each word that had been learned. After presentation of the target word for 2,000 ms and the grey blank screen for 1,000 ms, participants were instructed to press one button (“M”) if they wanted to remember the word or another (“V”) if they wanted to forget the word. Participants had unlimited time to make their choice. As in the forced-choice condition, each trial ended with a blank screen for 1,000 ms.

##### Study phase: Yoked forced-choice

**Fig. 1:**
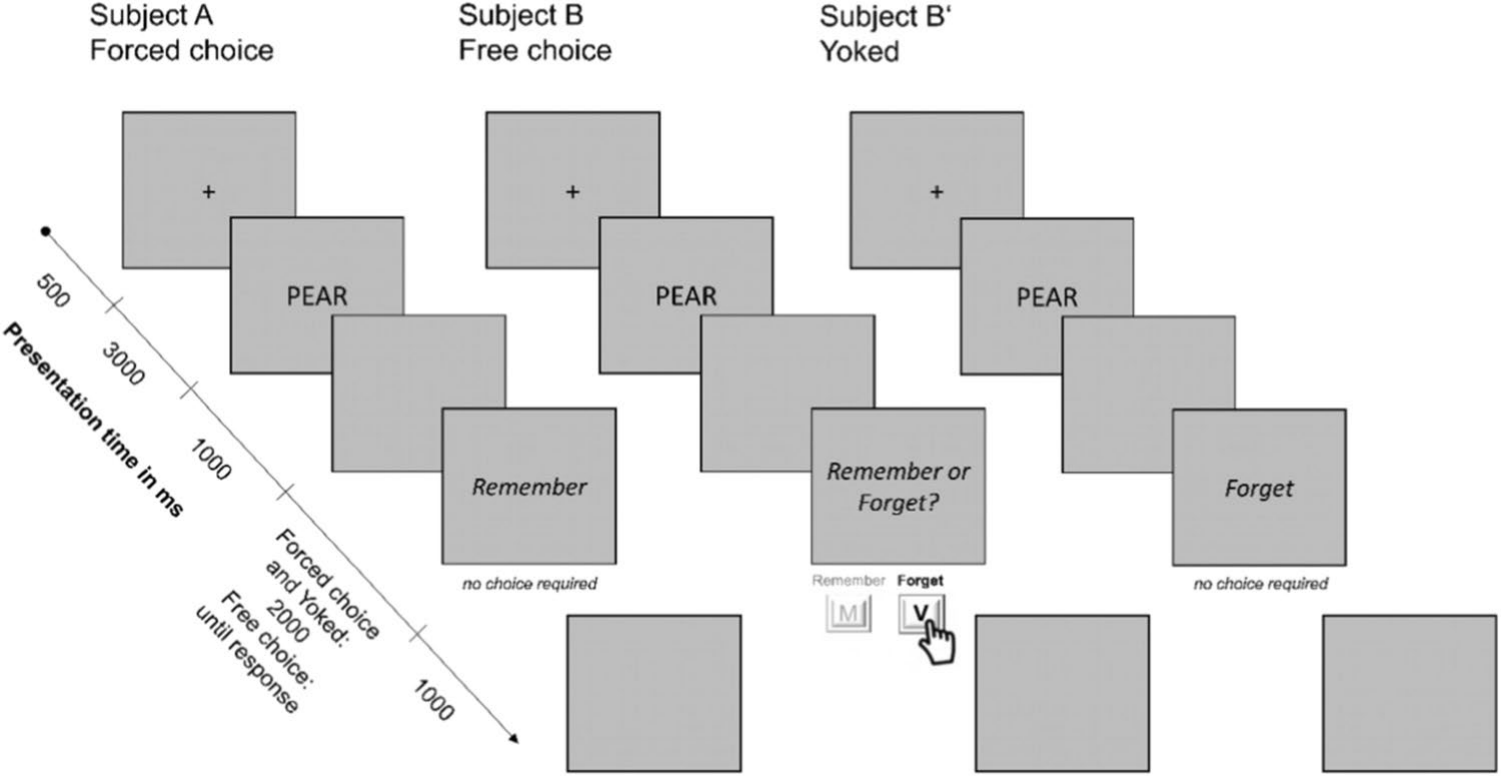
Visualization of the procedure in Experiment 1

The study phase for the yoked forced-choice condition was identical to that of the forced-choice condition, with the following exceptions. (1) The target words were not chosen randomly, but rather were yoked to the words presented to subjects in the free-choice condition. In other words, for each participant in the free-choice condition, a corresponding participant in the yoked forced-choice condition received the same target words, presented in the same order (though the presentation times, including cue presentation time, were the same as those in the forced-choice condition). (2) The cues were also not chosen randomly, but reflected the choices made by the corresponding participant in the free-choice condition. For example, if participant B in the free-choice condition saw the word APPLE and chose to forget this word, then participant *B’* in the yoked forced-choice condition also saw the word APPLE (in the same position of the list) and was instructed to forget this word (see Fig. 1 for an illustrated example).

##### Retention interval

Immediately following the study phase, participants were instructed that they would perform a series of addition and multiplication problems for 1 min. The participants were asked to type in the correct answer to the problem shown on the screen. The participants worked through as many problems as possible within 60 s. The task ended after an answer was entered after the 60-s mark (in other words, participants were always given time to complete the final task they were working on after the 60 s were up).

##### Test phase

Following the retention interval, participants were instructed to remember the words that they had seen in the study phase. However, they were now explicitly instructed to remember *all* of the words, both the words that they were instructed to/chose to remember and the words they were instructed to/chose to forget. Participants typed each word into the program and then pressed enter.^2^ They continued this process until they could not remember any further words, at which point they were instructed to press enter without writing any word. This ended the test phase.

##### Questionnaires and debriefing phase

At the end of the study participants were asked to answer three questions about how they completed the task (see the OSF page: https://osf.io/qfc2w/). Afterwards, participants were debriefed about the purpose of the experiment and the need for the deception (i.e., being told that some words should be forgotten, although they were ultimately tested). Finally, participants were given a code that could be used to obtain their study credits, were thanked for their participation, were given contact information if they had questions about the study, and were reminded how to upload the data.

### Results

All data were first processed using Microsoft Excel, before being analyzed in SPSS. An alpha level of .05 was used across all analyses, unless otherwise noted. For a visualization of the data-processing steps, details about the data processing, and access to the data, please visit the OSF page (https://osf.io/qfc2w/).

#### Recall performance

For determining correct recall, we decided to not exclude words that were incorrectly spelled, as long as the meaning was not changed. Two raters (RPMH and Svenja Eickemeier) determined accuracy independently and agreed on 100% of trials. We submitted the proportion of recalled words to a 3 (Condition: forced-choice, yoked forced-choice, free-choice) × 2 (Item type: remember, forget) mixed ANOVA, whereby Condition was a between-subjects and Item type a within-subjects factor.

**Fig. 2:**
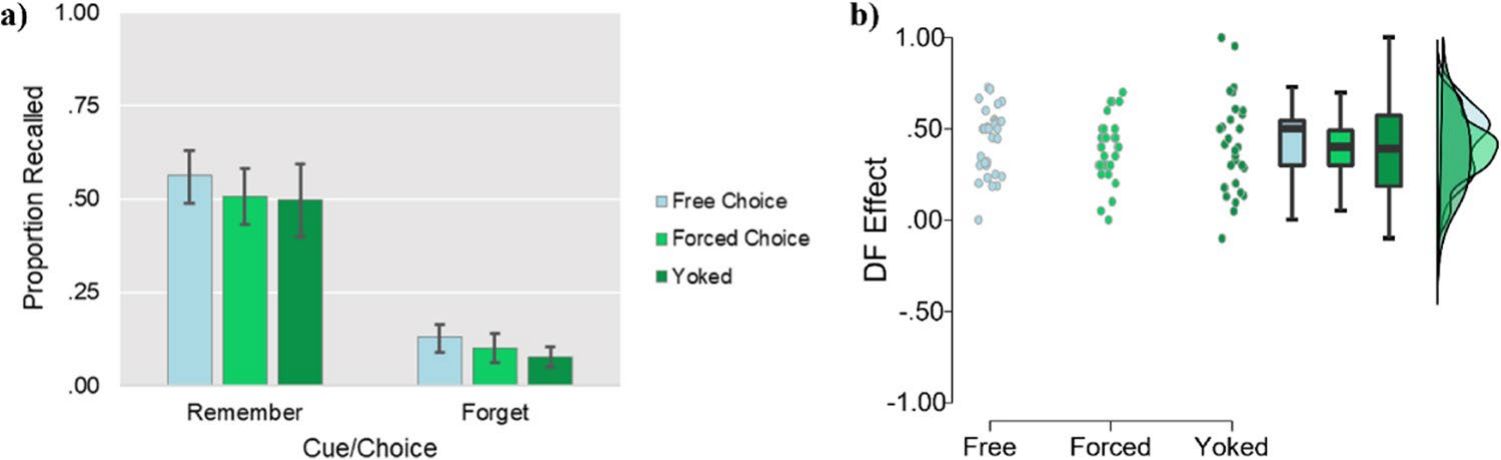
(**a**) Mean proportion of items recalled as a function of Condition and Item type in Experiment 1. Error bars represent 95% confidence intervals. (**b**) Raincloud plot for the size of the directed forgetting effect (recall of remember items minus recall of forget items) across conditions in Experiment 1

The ANOVA revealed a main effect of Item type, *F* (1, 87) = 342.08, *p* < .001, *η*_*p*_^*2*^ = .80, whereby the proportion of “remember” words recalled (M = .51, *SD* = .22) was greater than the proportion of “forget” words recalled (M = .10, *SD* = .10). Neither the main effect of Condition, *F* (2, 87) = 2.27, *p* = .109, *η*_*p*_^*2*^ = .05, nor the interaction, *F* (2, 87) = .33, *p* = .718, *η*_*p*_^*2*^ = .01, were significant. However, a Helmert contrast showed a higher proportion of words recalled in the free-choice condition (*M* = .35, *SD* = .12) than in the forced-choice and yoked forced-choice (*M* = .29, *SD* = .14) conditions combined, *p* = .040, but that there was no evidence of a difference between the forced-choice (*M* = .29, *SD* = .14) and yoked (*M* = .29, *SD* = .14) conditions, *p* = .668 (see Fig. 2 for an overview). Finally, paired-samples *t* tests indicated that the difference in recall between to-be-remembered and to-be-forgotten items was significant in each of the three conditions (forced choice: *t* (29) = 12.29, *p* < .001, *dz* = 2.24; free choice: *t* (29) = 12.98, *p* < .001, *dz* = 2.37; yoked: *t* (29) = 8.48, *p* < .001, *dz* = 1.58).^3^

#### Free-choice analyses

Given the novelty of our procedure, it was necessary to run several analyses to ensure that the instructions were being followed and to investigate how participants were completing the free-choice task.

##### Frequency of choices

**Fig. 3:**
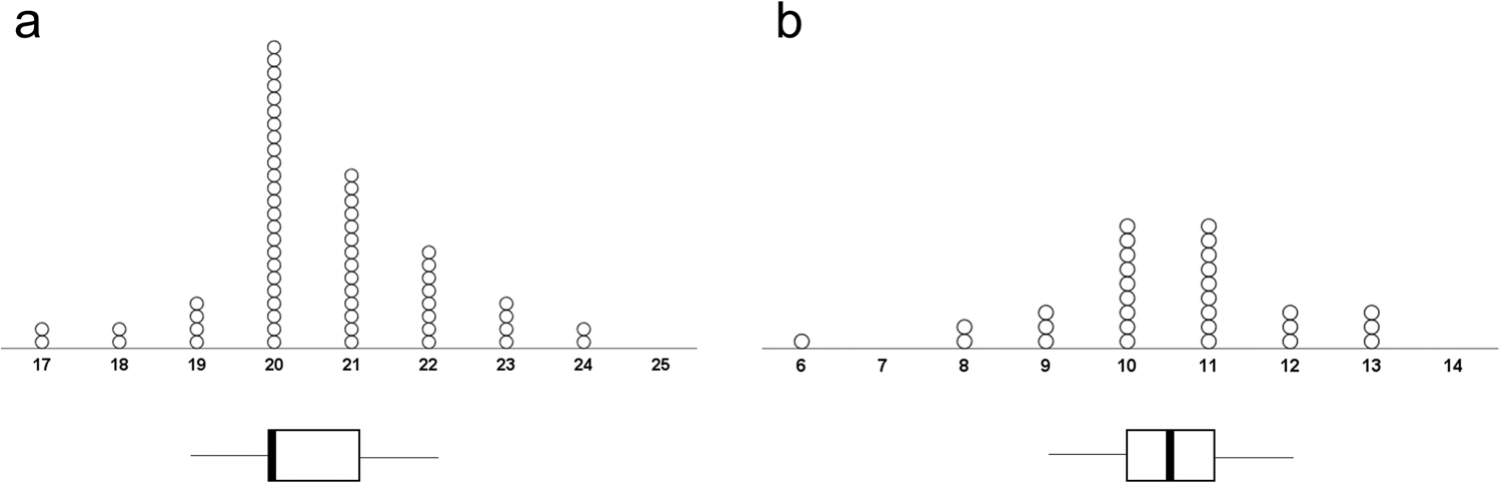
Frequency of remember choices in Experiment 1 (**a**) and in Experiment 2 (**b**). *Note:* The thick vertical lines in the boxplots in 3a and 3b represent the respective median of “choices to remember” (Experiment 1: *Mdn* = 20; Experiment 2: *Mdn* = 10.5). The desired number of remember choices was *n* = 20 in Experiment 1 and *n* = 10 in Experiment 2

In order to investigate whether participants could successfully follow the instructions and choose roughly half of the studied words to remember in the free-choice condition, we submitted the frequency of remember choices (see Fig. 3) to a one-sample *t* test against the value of 20. This *t* test revealed a significant difference, *t* (29) = 2.26, *p* = .031, *d* = 0.41, indicating that the mean number of remember choices (*M* = 20.60, *SD* = 1.45), was slightly larger than the desired count of 20. Inspection of Fig. 3 shows that, while most participants chose exactly half of the words to remember, there was a bias among the other participants to choose slightly more words to remember than to forget. We conducted an additional ANOVA for recall performance parallel to the one reported above, after excluding subjects who chose to forget more than half of the words. This ANOVA (n = 82; four subjects removed from the free choice and yoked conditions each), however, showed the same pattern of results as the one reported above, suggesting that somewhat irregular frequencies of choices were not the reason for our main findings (for a full description of this ANOVA, see “Additional analyses” on the OSF page).

##### Time to choose

A paired-samples *t* test revealed there was no evidence for a difference between the time to choose to remember a word (*M* = 1,517.06, *SD* = 1,513.40) and the time to choose to forget a word (*M* = 1,283.32, *SD* = 936.95), *t* (29) = 1.18, *p* = .249, *d* = 0.22.

### Discussion

In Experiment 1 we introduced a between-subjects variant of the free-choice procedure in IMDF. We replicated previous research in finding DF effects with the forced-choice task, and extended this research in finding DF effects with a free-choice task as well. Furthermore, there was no interaction between the Condition and the Item type, indicating that there were no significant differences found between any of the conditions relating to the directed forgetting effect. This seems to indicate that allowing participants to freely choose what to remember and forget does not lead to a greater amount of forgetting, in comparison to the forced-choice conditions. Also, while there was no main effect of Condition, a Helmert contrast did show superior recall performance in the free-choice condition than in either of the forced-choice conditions (see the *General discussion* for a discussion of the self-choice effect).

Despite attempting to control for item-selection issues in Experiment 1 by including the yoked forced-choice condition, a major potential flaw in the design still remained. Namely, participants in the free-choice condition may have decided to forget an upcoming word, without actually having learned it. While there was no direct evidence for this happening in the response times (see above), the possibility of this happening remained. If participants never learned the “forget” words, this would obviously severely decrease the ability to interpret the findings as evidence of voluntary forgetting. In order to address this problem, and to reduce the between-subjects variability between tasks, Experiment 2 was conducted with a within-subject design in which the type of task (forced- or free-choice) was varied on a trial-by-trial basis.

Another potential issue in Experiment 1 was the near-floor recall performance for ”forget” words. Therefore, in Experiment 2 we added a recognition test, in order to avoid floor effects. We expected to observe intact IMDF on both recall and recognition, since this is the typical finding in the literature (e.g., Basden et al., 1993; MacLeod, 1975).

## Experiment 2

### Method

#### Participants

For Experiment 2 we performed an a priori sample size calculation, with a slightly different rationale for gathering the sample as in Experiment 1. Given that we did not find clear evidence of a self-choice effect in Experiment 1, we based our sample size calculation on the ability to potentially find such an effect in Experiment 2. A recent multi-study paper estimated the effect size of the self-choice effect as *d* = .62 (Baldwin et al., 2021). Using the software program G*Power (Faul et al., 2007) to conduct our sample size calculation, we looked for the required sample size given a within-subject design focusing on the self-choice effect for the to-be-remembered words. Therefore, we used a paired-samples *t* test design, with one tail, an estimated effect of *d*_*z*_ = .62, and alpha and beta error rates of .05. Based on this calculation we needed *n* = 30 participants to find the effect. As can be found on our preregistration (https://osf.io/qfc2w/), we determined to collect data until we had 30 useable data sets.

Accordingly, 34 students (who did not take part in either Experiment 1 or any other directed forgetting experiment at the university) participated in return for partial course credit. Informed consent was obtained from all individual subjects included in the study. Four subjects were removed from the data set for not following instructions and always choosing “forget” in the free-choice task. The sample used for analyses included 30 subjects, (see Table 1 for demographic information) all but one of whom were students from the University of Hildesheim. The other participant was a student at a different university in Germany, Greifswald University.

#### Design

A 2 (trial type: forced-choice, free-choice) × 2 (item type: remember, forget) design was used. Both factors varied within subjects (please note: in the case of free-choice condition, the response was not varied but chosen by participants).

#### Material

The materials used were the same as in Experiment 1. However, in Experiment 2 only 40 words were chosen from the WWN database (Lahl et al., 2009) for the study phase. In order to try and improve overall recall performance, the 40 words chosen most frequently to be remembered by participants in the free-choice condition of Experiment 1 were used as the words for the study phase in Experiment 2. The 40 lures (i.e., new words) for the recognition test were comprised of the 40 words chosen least frequently to be remembered by participants in the free-choice condition of Experiment 1. The word list can be found on the OSF page: https://osf.io/qfc2w/. All other material was identical to Experiment 1.

#### Procedure

The procedure for Experiment 2 was identical to that of Experiment 1, except for the following differences. At study, all participants engaged in both forced- and free-choice trials. There was a total of 40 trials in the study phase. The order of trials varied randomly for each participant (note that the average serial position for each condition across subjects was similar: Forced/Remember (19.31), Forced/Forget (20.42), Free/Remember (21.31), Free/Forget (20.95)). The timing of the two trial types was identical to Experiment 1. A further difference to Experiment 1 was that, following the free-recall phase in Experiment 2, participants also completed an old/new recognition test. There were 80 trials (40 target and 40 lure trials) and the order of trials was determined randomly for each participant. On each trial, participants indicated whether the presented item was an old item from the study phase or a new item. The recognition test was self-paced, with no time limit. One further difference to Experiment 1 was the addition of several questions at the end asking participants about the strategies they used to remember or forget words as a function of trial type and several questions probing how participants made their choices on free-choice trials. A list of all questions asked can be found on the OSF page: https://osf.io/qfc2w/.

### Results

All data were first processed using Microsoft Excel, before being analyzed in SPSS. For a visualization of the data-processing steps, details about the data processing, and access to the data, please visit the OSF page (https://osf.io/qfc2w/).

#### Recall performance

For determining correct recall, we decided to not exclude words that were incorrectly spelled, as long as the meaning was not changed. Two raters (RPMH and Svenja Eickemeier) determined accuracy independently and agreed on 100% of trials. We submitted the proportion of recalled words to a 2 (Trial type: forced-choice, free-choice) × 2 (Item type: remember, forget) repeated-measures ANOVA.

**Fig. 4:**
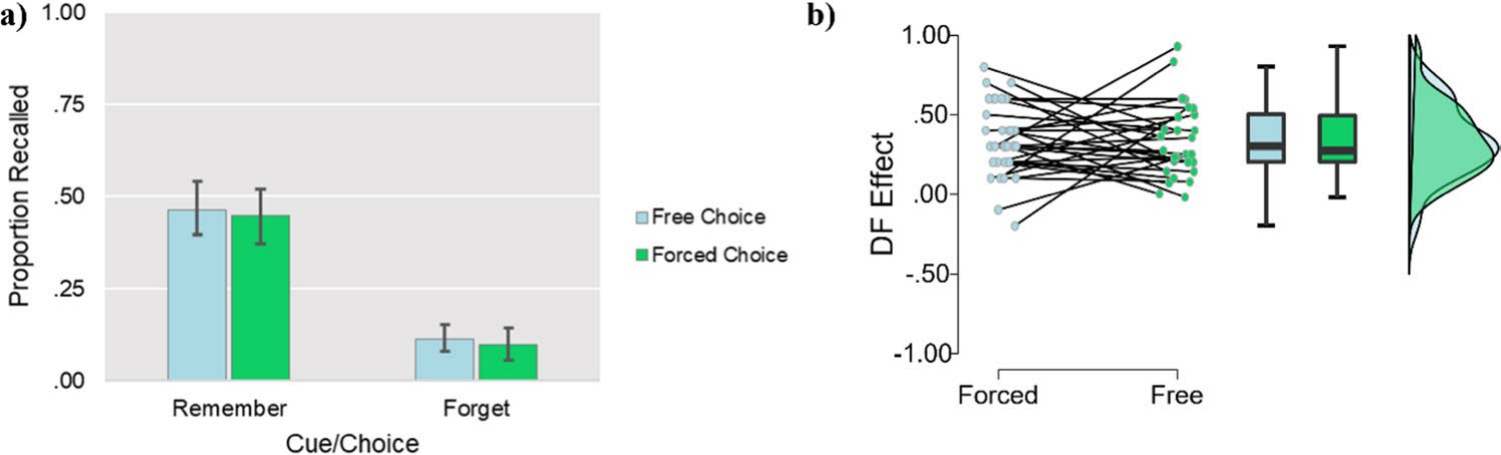
(**a**) Mean proportion of items recalled as a function of Condition and Item type in Experiment 2. Error bars represent 95% confidence intervals. (**b**) Raincloud plot for the size of the directed forgetting effect in recall (recall of remember items minus recall of forget items) across conditions in Experiment 2

The ANOVA revealed a main effect of Item type, *F* (1, 29) = 99.80, *p* < .001, *η*_*P*_^*2*^ = .78, whereby the proportion of “remember” words recalled (*M* = .46, *SD* = .19) was greater than the proportion of “forget” words recalled (*M* = .11, *SD* = .09). Neither the main effect of Trial Type, *F* (1, 29) = .56, *p* = .461, *η*_*P*_^*2*^ = .02, nor the interaction, *F* (1, 29) = .02, *p* = .905, *η*_*P*_^*2*^ < .001, were significant (see Fig. 4 for an overview). IMDF was intact for both trial types (forced choice: *t* (29) = 8.36, *p* < .001, *d* = 1.53; free choice: *t* (29) = 8.63, *p* < .001, *d* = 1.58).^4^

### Recognition performance

We report recognition performance as measured by *d*’.^5^ We used a log linear correction to address proportions of 0 and 1 (Hautus, 1995; Stanislaw & Todorov, 1999). We submitted corrected *d*’ scores to a 2 (Trial type: forced-choice, free-choice) × 2 (Item type: remember, forget) repeated-measures ANOVA. One extra participant was removed from the analyses for recognition performance, as they had an extremely high number of false alarms (proportion of false alarms = .53). Therefore, the data from 29 participants were entered into the recognition analyses.

**Fig. 5:**
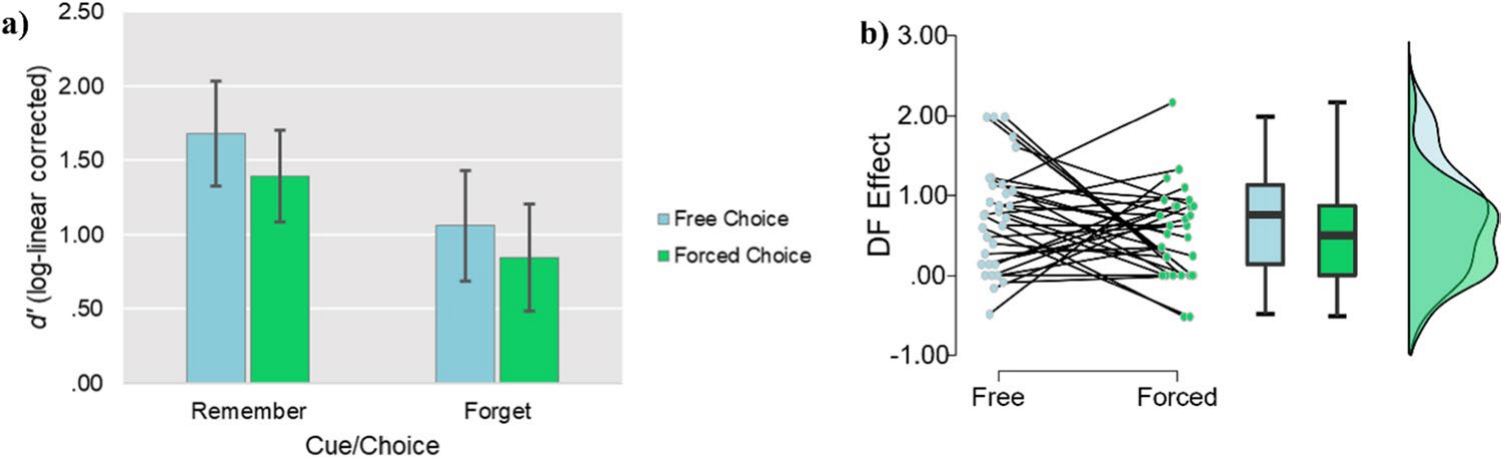
(**a**) Mean *d*’ as a function of Condition and Item type in Experiment 2. Error bars represent 95% confidence intervals. (**b**) Raincloud plot for the size of the directed forgetting effect on the recognition test (*d*’ for remember items minus *d*’ for forget items) across conditions in Experiment 2

Similar to recall performance, the ANOVA revealed a main effect of Item type, *F* (1, 28) = 63.32, *p* < .001, *η*_*P*_^*2*^ = .69, whereby *d*’ for “remember” words (*M* = 1.54, *SD* = .86) was greater than *d*’ for “forget” words (*M* = .95, *SD* = .97). Unlike for recall performance, there was also a main effect of Trial type, *F* (1, 28) = 11.65, *p* = .002, *η*_*P*_^*2*^ = .29, whereby *d*’ for freely chosen words (*M* = 1.37, *SD* = .96) was greater than *d*’ for forced-choice words (*M* = 1.12, *SD* = .88). There was no significant interaction between Trial type and Item Type, *F* (1, 28) = .20, *p* = .661, *η*_*P*_^*2*^ = .01 (see Fig. 5 for an overview). IMDF was again intact for both trial types (forced choice: *t* (28) = 5.24, *p* < .001, *d* = 0.97; free choice: *t* (28) = 6.02, *p* < .001, *d* = 1.12).^6^

Hit and false alarm rates can be found in Table 2. For recognition performance, we wanted to ensure that problems of both floor and ceiling performance were avoided. We checked for these problems by using one-sample *t* tests for the hit rates for forced- and free-choice “forget” words against guessing (i.e., .5) and for forced- and free-choice “remember” words against perfect performance (i.e., 1). All four *t* tests revealed that performance was above the guessing rate and below perfect performance, all *p* values ≤ .001.

**Table 2:** Mean (*SD*) loglinear corrected hit and false alarm rates from Experiment 2 as a function of Trial type and Item type

	Hit rate		False alarm rate
	Remember	Forget	
Forced-choice	.81 (.12)	.64 (.19)	.37 (.23)
Free-choice	.87 (.09)	.71 (.16)	

#### Free-choice analyses

As in Experiment 1, it was necessary to run several analyses to ensure that the instructions were being followed and to investigate how participants were completing the free-choice task.

##### Frequency of choices

In order to investigate whether participants could successfully complete the task of choosing roughly half of the free-choice words to remember, we submitted the frequency of remember choices (see Fig. 3) to a one-sample *t* test against the value of 10. This *t* test revealed no significant difference, *t* (29) = 1.54, *p* = .136, *d* = 0.28, indicating that the mean number of remember choices (*M* = 10.43, *SD* = 1.55) was not different than the desired count of 10. Inspection of Fig. 3 shows that most participants were able to complete the task perfectly or near perfectly. We conducted an additional ANOVA for recall performance parallel to the one reported above, after excluding subjects who chose to forget more than half of the words. This ANOVA (n = 24), however, showed the same pattern of results as the one reported above, suggesting that somewhat irregular frequencies of choices were not the reason for our main findings (for a full description of this ANOVA, and others with data from the recognition tests also showing the same pattern of results as above, see “Additional analyses” on the OSF page).

##### Time to choose

A paired-samples *t* test revealed there was no evidence for a difference between the time to choose to remember a word (*M* = 1,530.21, *SD* = 621.18) and the time to choose to forget a word (*M* = 1,817.84, *SD* = 1,098.45), *t* (29) = 1.71, *p* = .098, *d* = 0.31.

### Discussion

In Experiment 2 we extended the findings from, and addressed a major problem (i.e., the potential to not study to-be-forgotten words) inherent in, Experiment 1. Specifically, in Experiment 2, we introduced a within-subjects variant of the free-choice procedure in IMDF. One of the key improvements of Experiment 2 over Experiment 1 was that in Experiment 2 participants did not know whether a trial was going to be a free- or forced-choice trial, and therefore did not have the ability to make a “forget” choice before viewing the word. This ensures that all words were studied, and any DF effects were not simply a difference between remember and forget conditions in the likelihood to have initially encoded the word. We replicated previous research and Experiment 1 in finding DF effects in the forced-choice condition. In another extension of Experiment 1, we here found DF effects for the forced- and free-choice conditions for both free recall and old/new recognition. Also, similarly to Experiment 1, there were no significant differences found in the DF effects between the forced- and free-choice conditions, for either free recall or recognition. Taken together this seems to indicate that participants are able to forget words that they are instructed to forget, as well as words they voluntarily choose to forget, and that there is no significant difference in the degree of forgetting dependent on whether the forgetting is instructed or voluntary.

In Experiment 2, we failed to find evidence of a self-choice effect for free recall. This partially mirrors the results from Experiment 1, where there was no main effect of Condition, though the Helmert contrast did indicate that performance was better in the free-choice condition than the average across the forced-choice and yoked forced-choice conditions. That being said, we did find evidence of a self-choice effect for recognition performance in Experiment 2, with *d*’ scores generally indicating higher performance after free- than after forced-choice. We discuss the implications of our findings for the self-choice effect in the *General discussion*.

## General discussion

Researchers have long been interested in determining how much control humans have over their ability to remember and to forget. While research on remembering has investigated the influence of free-choice and agency, such as investigations into the self-choice effect (Monty & Perlmutter, 1975), nearly all previous research that has investigated the topic of voluntary forgetting has actually made use of a forced-choice paradigm, where participants were instructed as to which information to remember and which information to forget (e.g., Basden, 1996; for a review, see Hall et al., 2021). However, several investigations on the self-choice effect have provided indirect (Watanabe, 2001) or direct (Baldwin et al., 2021) evidence that humans do indeed have agency in actually forgetting the information which they choose to forget. Our research was mainly aimed at determining whether the IMDF was present when participants could freely choose which information to remember and forget, and to determine if there were statistically significant differences in IMDF as a function of whether the to be forgotten material was instructed or chosen.

### Voluntary forgetting

Experiment 1 of the current study used a between-subjects design while Experiment 2 used a within-subjects design. Despite the different methodologies used, there was much convergence in the results across the two studies. Most importantly, we showed evidence of voluntary, as well as instructed, directed forgetting. More specifically, subjects showed less of an ability to retrieve information they chose to forget than information they chose to remember. These results support the notion that information that individuals choose to forget is more difficult to retrieve at a later time.

Another key finding across the two experiments reported here is that there was no evidence that the size of the directed forgetting effect differed between the forced- and free-choice conditions. In other words, subjects showed better memory for to-be-remembered than to-be-forgotten words, and there was no indication of a difference in retrieval for the two types of words as a function of whether they were instructed to remember/forget the words or they chose to remember/forget the words. The size of the IMDF effect, in terms of Cohen’s *d*, was large in all cases, which is similar to findings in previous experiments (Hall et al., 2021).

It is important to mention that participants in both experiments were able to follow instructions and choose roughly half of the words to remember in the free-choice conditions. Furthermore, the debriefing questions at the end of Experiment 2 (see Appendix for a summary; see the OSF page for the full data) revealed that most of the participants believed the instructions that they only needed to remember the to-be-remembered words, and that they did not try to remember the to-be-forgotten words until specifically asked to do so in the retrieval phases. Indeed, none of the participants chose the most extreme value on the Likert scale which would have indicated they believed they needed to also remember the to-be-forgotten words. In line with our pre-registration, we therefore decided not to remove any subjects from the main analyses. However, additional analyses (which can be found under the “Additional analyses” on the OSF page) revealed that removing subjects who reported a value below the midpoint of the scale, indicating they tended to believe they must also remember to-be-forgotten words, did not influence the pattern of results in either Experiment 1 or Experiment 2.

In fact, recall of to-be-forgotten words was very low in both our experiments, despite participants being explicitly encouraged to recall all words that they had studied beforehand. Recall performance in IMDF can vary greatly across studies, and similarly low recall levels were also observed in prior work (e.g., Basden et al., 1993; MacLeod, 1989; Woodward et al., 1973). Nevertheless, we included an additional recognition test in Experiment 2 to avoid floor levels. As expected, performance on this recognition test was clearly above floor levels, and again similar to performance levels observed in prior work (e.g., Bancroft et al., 2013; Basden et al., 1993; Taylor et al., 2018). Most importantly, our main findings generalized across test formats, suggesting that they did not hinge on floor-level performance for to-be-forgotten words. An issue to note is, however, that due to the COVID-19 pandemic, our experiments were conducted remotely instead of in the laboratory. We cannot exclude the possibility that participants’ motivation for engaging in effortful memorization and recall of single, unrelated words was reduced with such remote data collection. At least potentially, this could explain the relatively low performance levels observed in our experiments.

Taken all together, our results suggest that both voluntary and instructed forgetting are possible and that the size of DF effects do not seem to differ as a function of the choice. Furthermore, due to the control we took, our findings are likely not due to item-selection issues, and/or subjects choosing to forget before actually viewing the to-be-studied words. Our results, however, are not only interesting for their implications for voluntary forgetting.

## Self-choice effect in memory

Across two experiments, we failed to find strong evidence of the frequently found self-choice effect in free recall (e.g., Baldwin et al., 2021; Takahashi, 1992, 2002). In Experiment 2 we found no evidence of a self-choice effect in free recall. In Experiment 1 we did find weak evidence of a self-choice effect in free recall, in that the Helmert contrast showed that the free-choice condition performed better than the average of the forced-choice and the yoked-forced-choice conditions. However, in the ANOVA there was no main effect of condition. That being said, supporting previous research that found a self-choice effect with recognition tests (e.g., DuBrow et al., 2019; Kuhl & Kazén, 1994; Murty et al., 2015), we did find higher *d*’ scores in the free- than forced-choice condition in Experiment 2 on the recognition task. Our results, therefore, should be seen as providing mixed support for the presence of a self-choice effect.

If we consider the self-choice effect in Experiment 2 on the recognition test, it is interesting to note that no significant interaction was found. In other words, there was superior recognition of free-choice than forced-choice “remember” words as well as “forget” words. This is interesting, as it mirrors results from a study by Watanabe (2001) in which participants also had higher recognition rates for unchosen (similar to “forget” words in the current study) as well as chosen (similar to “remember” words in the current study) items in free-choice than forced-choice conditions (see also Takahashi, 1991; Toyota, 2013).

Though not the focus of this paper, the finding of a self-choice effect for both “remember” and “forget” items is in line with the multiple-cue hypothesis adapted by Watanabe (2001). The hypothesis was originally proposed in the context of the generation effect (Soraci et al., 1994, 1999) and suggested that multiple alternative response options are activated during generation, which then act as effective retrieval cues at test. Based on this prior work, Watanabe (2001) argued that encoding processes were involved in the act of choosing. In order to be rejected, contents first have to be processed. In other words, more elaborative processing may occur with free- than forced-choice. If choosing does lead to more elaboration for both the to-be-remembered and to-be-forgotten words, then there should be greater memory for both the remember and forget words in the free- than forced-choice condition.

This pattern of results would not be predicted by other theories that have been used to explain the self-choice effect, such as a motivation theory (Monty et al., 1973), which would likely predict that participants are not very motivated to remember items that are chosen to be forgotten, or a meta-memory theory (Takahashi, 1991), which would likely predict that participants choose to forget words that are particularly difficult to remember.

## Forced- versus free-choice research more broadly

Research on the differences between forced and free choices has long been of interest to cognitive psychologists (Berlyne, 1957). Indeed, in addition to finding differences in response times on forced- and free-choice tasks, possibly because of differential time needed for evidence accumulation in favor of a response option (Naefgen et al., 2018), forced- and free-choice tasks have also been shown to differentially influence stimulus-response binding (Pfister et al., 2010). That being said, there is a growing body of evidence showing that forced- and free-choice tasks do not differentially influence a range of cognitive effects. For example, the pattern of the negative compatibility effect is similar on both forced- and free-choice response priming tasks (Bermeitinger & Hackländer, 2018), dual-task costs (using a secondary forced-choice task) are similar for forced- and free-choice tasks (Jancyzk et al., 2015), and that both forced- and free-choice secondary tasks lead to similar costs for a forced-choice primary task, indicating that they activate and rely on similar cognitive representations (Richardson et al., 2020).

The findings from our two experiments mostly align with the recent trend of finding similar patterns for both forced- and free-choice tasks. While we did find evidence of a self-choice effect on the recognition test in Experiment 2 (discussed above), across both experiments we did not find that the IMDF size differed as a function of Trial type (i.e., whether it was a forced- or free-choice trial). To be more precise, we found that forgetting occurred to the same degree when subjects were instructed to forget an item or when they chose to forget an item, in both a between-subjects design (Exp. 1) and a within-subject design (Exp. 2).

One limitation of not only the present work, but most research on free versus forced choice in lab settings, is that participants, even when given free choice, still follow task instructions. For example, in the present study, participants freely chose to forget or remember specific words, but only did so to comply with the experiment. To what degree their choice was fully free can be debated. Although we would argue that the differentiation between free and forced choice in lab contexts is still meaningful, completely free choice in real-world settings could differ from and is likely more complex than the instances of free choice that can currently be captured in the lab.

## Future directions

The methods developed in the two experiments here can be used in the future to answer research questions related to voluntary forgetting and the role of choice in memory. Most directly, one question of interest that can be investigated with the free-choice methodology used here is what motivates people to forget some information, or put differently, what types of information do people try to forget voluntarily? Idiosyncratic preferences by individuals might be particularly interesting, but also difficult to examine. Another question of interest is how the choice to forget influences the subjective feelings about the target (i.e., to be forgotten) information and subjective feelings of agency related to remembering and forgetting.

One more question of interest that could potentially be investigated with a free-choice IMDF paradigm concerns the resources used for remembering and forgetting. For example, free-choice IMDF tasks may be more cognitively demanding or require more evidence accumulation (Naefgen et al., 2018) than forced-choice IMDF tasks. Furthermore, there is a question as to whether, within a free-choice paradigm, choosing to remember is equally, more, or less cognitively demanding than choosing to forget. To date, this “choice” has been investigated using the standard instructed forgetting paradigm (e.g., Fawcett & Taylor, 2008; Tan et al., 2020). It could be that using a free-choice IMDF paradigm will illuminate differences in the resources required to choose to remember or forget, which may differ from an instructed forgetting task.

It should also be noted that, while we found no evidence of differences between the free- and forced-choice conditions, in terms of the directed forgetting effect, in the current experiments, it is possible that different processes led to similar forced and free choice directed forgetting effects. For example, it could be that subjects chose to forget words that were simply difficult to remember. While we may have expected larger self-choice effects if this were the case, the possibility still remains. One way to address this in future research could be to specifically vary item memorability (e.g., provide a mix of concrete vs. abstract words, or also of neutral vs. negative words). If participants indeed tend to choose information that is difficult to remember, they should predominantly choose to forget abstract or neutral information, which might in turn also produce differences in the magnitude of the observed forgetting. In any case, expanding on the paradigm presented here, one goal for future research should be to more deeply investigate the processes underlying the forced and free choice directed forgetting effects and to attempt to determine whether they are indeed the same.

## Conclusion

In support of recent research (Baldwin et al., 2021; Watanabe, 2001) and long-held notions about voluntary forgetting, our experiments showed that humans can forget the information that they choose to forget, in addition to the information that they are instructed to forget. Furthermore, our research shows that the degree of forgetting does likely not differ as a function of whether the to be forgotten material was instructed or voluntarily chosen. Future research using the procedures developed here can further investigate both similarities and dissimilarities between instructed and voluntary forgetting, including subjective aspects related to the experience of remembering and forgetting.

## Figures and Tables

**Fig. 6:**
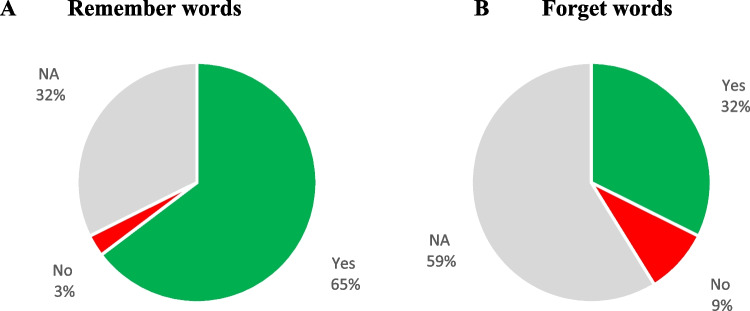
Proportions of participants who used/did not use a strategy.

## Data Availability

The data that support the findings presented in this paper are openly available on the Open Science Framework at https://osf.io/qfc2w/. Experiment 2 was preregistered on the Open Science Framework and can be found at osf.io/4xhzn.

## Appendix

**Debriefing questions**

After completing Experiment 2, participants were asked several questions with regard to, for example, the compliance, the sense of control during the experiment, and the strategies used while trying to remember or forget a given word. Table 3 summarizes the participants’ ratings.

In general, it seems, based on their ratings, that participants believed the instruction that they were to remember only the words followed by a “remember” cue or that were chosen freely to be remembered. In the same vein, participants indicated that they did not try hard to remember to-be-forgotten words. Hence, they were also not confident in their performance of remembering to-be-forgotten words in comparison with remembering to-be-remembered words.

Participants reported having slightly more control over the process of forgetting when they were given the opportunity to choose to forget a word as compared with when they were cued to forget a word. In addition, participants indicated that the process of forgetting was more automatic (less conscious) for words they had chosen to forget than when they were cued to forget a word.

We also asked the participants if they used strategies in order to remember or forget a word (see Fig. 6). Note that 32 % (remember words) and 59% (forget words) of the participants did not respond to these questions, respectively. It remains unclear whether these participants, or a subset thereof, actually did not have a strategy to either remember or forget words. However, when turning to those participants who actually responded to these questions, we may conclude that more participants indicated to have used a strategy for remembering words as compared with forgetting words.

Further questions aimed at the strategies in more detail. Participants were asked to describe their strategy/strategies for remembering words in each condition (Table 4). Participants were also asked to describe how they chose which words to remember or forget in the free choice condition (Table 5).

Based on the participants’ answers, the strategies for remembering to-be-remembered words do not seem to be different as a function of whether the choice was cued or freely made. In general, the most often reported strategies were deep encoding strategies (“making up a story” out of the to-be-remembered words, mnemonics/forming associations, visualization of words) and the active rehearsal by repeating the to-be-remembered words either silently or loudly.

When looking at the decisions for remembering or forgetting a word in the free choice condition, there are also no apparent differences in the reported reasons for the respective choice. Most often participants reported to have chosen to remember words that fit a made-up story, while they rejected to remember words that did not fit their story. Likewise, some participants responded to have chosen to remember those words that were more easily associated with the other words they had chosen to remember. Only one participant indicated to have chosen words based on their length (remember short words, forget long words), while one participant had tried to memorize only the second half of the presented words.

**Table 3:** Participants’ (*N* = 24) ratings on a 7-point Likert scale

Question	Lower pole (= 1)	Upper pole (= 7)	*M*	*Mdn*	*SD*
During the learning phase, how much did you believe that you only needed to remember the words that were followed by a "REMEMBER" cue, or those that you chose as "to remember"?	Not at all	Very much	6.08	6.50	1.18
During the learning phase, how much did you try to remember words that were followed by a "FORGET" cue or words that you chose as "to forget"?	Not at all	Very much	1.96	2.00	1.30
How good do you think you were at remembering the “FORGET” cued words or the words you chose as “to forget” in comparison to the “REMEMBER” cued words or the words you chose as “to remember”?	Much worse	Much better	2.46	2.00	1.14
How much did you feel you could control the process of forgetting, when you were cued to forget a word?	No control	Complete control	4.21	4.00	1.62
How much did you feel you could control the process of forgetting, when you chose to forget a word?	No control	Complete control	4.83	5.00	1.34
How automatically or consciously did the forgetting occur, when you were cued to forget a word?	Completely automatically	Completely consciously	4.29	4.50	1.46
How automatically or consciously did the forgetting occur, when you chose to forget a word?	Completely automatically	Completely consciously	3.75	3.50	1.62

**Table 4:** Frequencies of used strategies for remembering words in each condition.

Condition *(n)*	Making up a story	Mnemonics/ associations	Active rehearsal/ repetition	Visualization of words	Stringing words together
Remembering, cued (16)	7	2	7	2	1
Remembering, free choice (17)	7	5	5	3	-

*Notes:* Due to a programming error only part of the data of participants who had responded to have used a strategy could be processed. Participants were allowed to report multiple strategies per condition

**Table 5:** Frequencies of used strategies for the decision to remember/forget a word

Question *(n)*	Fit to made up story	Associations	Categorization	Choice based on word length	Forget first half, remember second
How did you decide which words to remember? (14)	7	4	1	1	1
How did you decide which words to forget? (13)	5	4	2	1	1

*Notes:* Due to a programming error only part of the data of participants who had responded to have used a strategy could be processed. Participants were allowed to report multiple strategies per condition

## Footnotes

1 We specifically focus on IMDF in this article, which should not be confused with the related paradigm of list-method directed forgetting. For more information on list-method directed forgetting, see reviews by Bäuml et al. (2020), MacLeod (1998), and Sahakyan et al. (2013).

2 E-Prime uses a QWERTY keyboard, which differs from standard keyboards in Germany. Participants were instructed to use common substitutions for common characters, e.g., OE instead of Ö, and to pay attention to the fact that the Y and Z were reversed.

3 These values for Cohen’s *d* may suggest a somewhat smaller IMDF effect in the yoked condition relative to the free-choice condition. An additional ANOVA directly contrasting these two conditions, however, also revealed no significant interaction effect between condition and item type, *F* (1, 58) = .22, *p* = .644, *η*_*p*_^*2*^ = .004. Furthermore, Bayesian analyses using JASP revealed moderate or anecdotal evidence in favor of the null hypothesis for both a comparison in the DF effect between the free-choice and yoked condition recall BF_10_ = .29 and the free-choice and the forced-choice condition BF_10_ = .38. See the OSF page for a full description of the Bayesian analyses.

4 A Bayesian paired-samples t test using JASP revealed moderate evidence in favor of the null hypothesis, that there is no difference in the DF effect between the forced- and free-choice trials, BF_10_ = .20.

5 Analyses using the hit rate closely mirror those using *d*’, and so are not presented here. These analyses can be found on the “Additional analyses” on the OSF page.

6 A Bayesian paired-samples *t* test using JASP revealed moderate evidence in favor of the null hypothesis, that there is no difference in the DF effect between the forced- and free-choice trials, BF_10_ = .22
